# Knowledge, attitude and support for exclusive breastfeeding among bankers in Mainland Local Government in Lagos State, Nigeria

**DOI:** 10.1186/s13006-018-0182-9

**Published:** 2018-08-22

**Authors:** Osiyosola O. Osibogun, Tolulope F. Olufunlayo, Samson O. Oyibo

**Affiliations:** 1 Department of Community Health and Primary Care, College of Medicine, University of Lagos, Lagos, Nigeria; 2 Department of Medicine, Peterborough City Hospital, Bretton Gate, Peterborough, PE3 9GZ UK

**Keywords:** Breastfeeding, Knowledge, Workplace-based support, Cross-sectional survey, Baby milk

## Abstract

**Background:**

Breastfeeding is a recognized means of ensuring optimal nutrition for the infant. Exclusive breastfeeding is defined as feeding an infant child breast milk only, and for optimal nutrition it is recommended that infants be exclusively breastfed for the first 6 months of life. Without workplace support, exclusive breastfeeding is difficult for working mothers who return to work. The aim of this study was to examine the knowledge, attitude, and support for exclusive breastfeeding among female bank workers in Lagos, Nigeria.

**Methods:**

This was a cross-sectional descriptive study. A total of 210 mothers from all banks in Mainland Local Government, Lagos State were selected using systematic sampling, with a response rate of 95%, so we had 200 respondents. A pretested, structured, self-administered questionnaire adapted from previous studies was used for data collection.

**Results:**

The mean age of the respondents in this study was 33 years (range 21- 50 years). Of the 200 respondents 188 (94%) had a good knowledge of exclusive breastfeeding and 173 (86.5%) of them reported that the hospital was their source of information. More than 180 (90%) respondents had a positive attitude towards exclusive breastfeeding. Exclusive breastfeeding was practiced by 112 (56%) of the respondents, however, only 57 (28.5%) practiced exclusive breastfeeding for up to 6 months postdelivery. The major source of breastfeeding support came from the baby’s father for 88 (44%) respondents while the least breastfeeding support came from the workplace for three (1.5%) respondents. The duration of maternity leave was mostly 3 months, and less than 20 (10%) respondents reported having support for breastfeeding at the workplace.

**Conclusions:**

In spite of the good knowledge of exclusive breastfeeding among the respondents as well as their positive attitude, the exclusive breastfeeding rate up to 6 months postpartum was very low. This may be related to the inadequate breastfeeding support from the various support systems especially in the workplace, as demonstrated in this survey. There should be advocacy for a government policy for a mandatory 6 month maternity leave for all working mothers.

## Background

The National Demographic and Health Survey (NDHS) 2013 study in Nigeria showed that while almost 70 % of children 0–23 months are predominantly breastfed (breast milk and only plain water or non-milk liquids such as juice and other liquids), only 17 % (17%) of children under age 6 months are exclusively breastfed [1]. The aforementioned partially explains the high incidence of infant malnutrition and mortality experienced in developing countries which is mainly due to poor infant feeding practices [2, 3].

Exclusive breastfeeding (EBF) is defined as feeding an infant child breast milk only. For optimal nutrition, it is recommended that a child be breastfed exclusively up to 6 months. As a result of rapid changes in the socio-cultural and economic situations worldwide, particularly the rapid urbanization and developing processes going on in developing countries, and the increasingly harsh economic environment, the need for income producing activity of women has increased. The educational and occupational status of the mother is an attribute that help determine the time allocation for women and children [4]. Given the numerous benefits of breastfeeding it is important that employed women understand its importance and are given the required support at every level.

There is lack of data regarding the number of Nigerian women employed within 6 months following birth. The duration of statutory maternity leave for working mothers in Nigeria is only 3 months, and support such as facilities for breast milk pumping and storage in the workplace are not widely available. An unfavourable working environment can make it difficult for mothers to implement and continue exclusive breastfeeding [5]. In recognition of the problems faced by working mothers in practicing exclusive breastfeeding, the World Breastfeeding Week 2015 adopted the theme, “Breastfeeding and work: let’s make it work”, to garner support from all sectors to enable women worldwide to work and breastfeed successfully [6].

There is paucity of data related to support systems for breastfeeding mothers in the workplace in Nigeria. A study among female resident doctors in a tertiary institution in Nigeria found the most important barrier to exclusive breastfeeding of their infants to be return to work (61% of respondents) [7]. A mixed methods study among breastfeeding mothers in Southwest Nigeria identified barriers to breastfeeding to include: perceived hunger after feeding baby (29%), maternal health problems (27%), fear of infant addiction to breast milk (26%), breast pains (25%), pressure from mother-in-law (25%), and return to work/business (24%) [8]. A mixed methods study in three Nigerian cities found return to work to be an important barrier to exclusive breastfeeding [9]. The aim of this study was to examine the knowledge, attitude, and support for exclusive breastfeeding among female workers in Nigeria.

## Methods

### Background of study area

Lagos State popularly known as the “Centre of excellence” was created in May 27 1967. It is located in the South Western part of the federation and shares boundaries with Ogun State both in the North and East and on the West by Republic of Benin. In the South, it stretches for 180 km along the coast of the Atlantic Ocean and occupies an area of 3,577sq km, 22% or 787sq km which consists of lagoons and creeks. It is a commercial nerve centre for all Nigerians and it is domiciled predominantly by Nigerians of different cultures and backgrounds [10]. The state is divided into administrative units called local government areas (LGAs). Mainland Local Government Area is one of twenty LGAs in Lagos state.

### Study population

This study was carried out among mothers working in commercial banks within Mainland Local Government area of Lagos State. Female bankers were chosen for this study because of their inflexible and demanding work schedules, which makes them vulnerable to not practising breastfeeding. There are 44 medium scale banks in Mainland local government. From a fact-finding visit before the commencement of the study, it was discovered that an average of 10 mothers worked in each bank. Therefore, the study population was estimated to be 440 (44 × 10). Only women who were working in the banks and had given birth to at least one child were included in this study.

### Sample size

We estimated that 67% of the study population practised exclusive breastfeeding based on the response to a previous breastfeeding survey carried out in another region of Nigeria [11]. Using the 95% confidence level and a 5% margin of error the calculated sample size for our survey was estimated to be 190 respondents. We made a 10% allowance for non-response making our minimum sample size 210 (two hundred and ten).

### Sampling method

The minimum sample size in this study (210) was estimated to be about half of the estimated population size (440) therefore by systematic sampling method every 2nd name on the list of names in the sampling frame of each bank was selected to participate in this study until the desired sample size was reached, the first respondent from the list was chosen by simple random sampling (balloting). Verbal invitations were made to potential participants and consent received before issuing questionnaires.

### Data measures

A structured, self-administered questionnaire adapted from several templates used in previous studies for mothers’ knowledge, attitude, practices and support systems for EBF was used to collect data [12–15]. We are unaware of previous questionnaires specific to workplace support systems so we formulated questions based on a previous survey [16].

### Data collection

The questionnaire was divided into five parts which are Section A: sociodemographic data, Section B: data regarding the knowledge of exclusive breastfeeding, Section C: data on the attitude of respondents towards the practice of EBF, Section D: data on the practice of respondents towards EBF and Section E: data on support systems towards exclusive breastfeeding among respondents. A five-point Likert scale (1 = “Strongly Agree”, 2 = “Agree”, 3 = “Indifferent”, 4 = “Disagree”, 5 = “Strongly Disagree”) was used to collect data for Section C (attitude towards EBF).

To ensure data quality, the questionnaire was pretested among ten female bankers in another Local Government within Lagos state (Mushin) and necessary modifications were made. The data collection was carried out over a period of 5 weeks by the researcher, and questionnaires were collected a few hours after distribution.

### Data analysis

Data was entered and analysed using Epi Info™ 7. Results were presented as frequency tables and charts as appropriate.

For Section B a correct answer to each question was given a score of one point while a wrong answer was given a score of zero. The total score for each respondent was calculated by summing up all the awarded marks and these were converted to percentages. A knowledge grade was assigned to each respondent based on his total percentage score. Knowledge score of 0–49% represented poor knowledge, 50–74% represented fair knowledge and a score of ≥75% represented good knowledge [4].

For Section C concerning attitude towards EBF, the responses on the Likert scale were given marks 1–5, respectively. The higher marks (4 & 5) represented a more positive attitude. The total attitude score for each respondent was calculated by adding all the awarded marks for all statements. This score was converted to a percentage (> 66% indicating a positive attitude and ≤ 66% indicating a negative attitude towards EBF).

### Ethical issues

Ethical approval was obtained from Health Research and Ethics Committee of the Lagos University Teaching Hospital in Lagos, Nigeria before collection of data. Informed verbal consent was obtained from participants, who were assured of strict anonymity and confidentiality during this survey.

## Results

A total of 210 questionnaires were distributed and of this 200 were returned and subsequently analysed giving a response rate of 95%.

Table 1 shows the baseline sociodemographic characteristics for the respondents. Their mean age (range) was 33 (21–50) years. Most (82%) of the respondents were Christians and majority (66%) belonged to the Yoruba ethnic group. Almost all (96.5%) of them were married and about three quarters of the respondents had either 1 or 2 children (the rest had 3 or more children). Most (58.5%) were junior staff of the bank. Almost all (96.5%) the husband/babies’ fathers had tertiary education and close to three-quarters of the babies’ fathers were senior professionals.

**Table 1:** Sociodemographic characteristics of respondents (*n* = 200)

Variable	Frequency	Percentage (%)
Age (years)		
21–30	70	35.5
31–40	118	59.0
41–50	12	6.0
Religion		
Christianity	164	82.0
Islam	36	18.0
Ethnic group		
Yoruba	132	66.0
Ibo	56	28.0
Hausa	5	2.5
Others	7	3.5
Marital status		
Single	5	2.5
Married	193	96.5
Widowed	2	1.0
Number of children		
1	64	32.0
2	91	45.5
3	40	20.0
4	5	2.5
Job level		
Executive staff	41	20.5
Junior staff	117	58.5
Managerial staff	42	21.0
Spouse’s level of education		
Primary	1	0.5
Secondary	6	3.0
Tertiary	193	96.5
Occupation of husband/partner		
Senior Professional	141	70.5
Intermediate Professional	51	25.5
Junior Professional	2	1.0
Semi-skilled	4	2.0
Unskilled	2	1.0

### Knowledge

Table 2 shows respondents’ knowledge of exclusive breastfeeding. All respondents had heard of EBF. More than three-quarters (77.5%) of the respondents knew that babies should be exclusively breastfed for the first 6 months of life while 3.5% were unsure. A large number (89–96%) of respondents were aware of the benefits of EBF to the infant. More than two-thirds (67%) of the respondents believe that breastfeeding should be on demand. The overall knowledge concerning EBF was good for 94% of respondents and fair for 6% of respondents. Additionally, most (95.5%) of the respondents had a positive view about expressing their breast milk with a breast pump or bare hands.

**Table 2:** Knowledge of exclusive breastfeeding (*n* = 200)

Variable	Frequency	Percentage (%)
Ever heard of exclusive breastfeeding		
Yes	200	100.0
Source of information		
Hospital	173	86.5
Friends	83	41.5
Family members	64	32.0
Media	71	35.5
Exclusive breastfeeding is:		
Giving baby breastmilk only	198	99.0
Giving baby breastmilk and water only	2	1.0
Recommended duration of exclusive breastfeeding is:		
6 weeks	11	5.5
3 months	14	7.0
4 months	4	2.0
6 months	155	77.5
9 months	9	4.5
Not sure	7	3.5
Knowledge of benefits of exclusive breastfeeding	Yes	
Breastmilk contains the complete food for the baby for the first 6 months of life	183	91.5
Giving the baby breastmilk alone provides enough energy to the baby for the first 6 months of life	181	90.5
Giving the baby breastmilk alone for the first 6 months helps to prevent the baby from getting infections	191	95.5
Giving the baby breastmilk alone for the first 6 months helps to prevent diarrhoea in babies	188	94.0
Exclusive breastfeeding help in reducing the number of children dying in Nigeria every day	179	89.5
Breastfeeding should be given on demand	134	67.0

### Attitude

Table 3 shows the attitude of respondents towards exclusive breastfeeding. The majority of respondents (61–95%) either disagreed or strongly disagreed with all ten statements. Ninety-five percent of respondents disagreed with the statement that breastfeeding is old fashioned, embarrassing and should not be done in public, two-thirds of respondents disagreed with the statement that it is okay to stop breastfeeding before the baby is 6 months old if their work takes them away from their baby most of the time, 61.5% of respondents disagreed with the statement that it is not okay to store expressed breast milk for their baby when they are not available. Almost all respondents (94.5%) demonstrated a positive attitude towards exclusive breastfeeding.

**Table 3:** Attitude of respondents towards exclusive breastfeeding (*n* = 200)

Statement	Strongly agree	Agree	Indifferent	Disagree	Strongly disagree
	*n* (%)	*n* (%)	*n* (%)	*n* (%)	*n* (%)
Exclusive breastfeeding is not important	10 (5.0)	9 (4.5)	2 (1.0)	23 (11.5)	156 (78.0)
Breastfeeding is old fashioned, embarrassing and should not be done in public	2 (1.0)	3 (1.5)	5 (2.5)	20 (10)	170 (85)
It is okay to stop breastfeeding before the baby is 6 months if my work takes me away from my baby most of the time	10 (5)	39 (19.5)	21 (10.5)	51 (25.5)	79 (39.5)
It is not okay to store expressed breast milk for my baby when I am not available	14 (7)	40 (20)	23 (11.5)	59 (29.5)	64 (32)
Breastfeeding affects care of other family members and marital relationship	2 (1)	7 (3.5)	11 (5.5)	51 (25.5)	129 (64.5)
I would not advise other mothers to practice exclusive breastfeeding	5 (2.5)	5 (2.5)	13 (6.5)	29 (14.5)	148 (74)
The benefits of breast milk lasts only until complementary feeding is introduced	7 (3.5)	20 (10)	14 (7)	68 (34)	91 (45.5)
Infant formula-feeding is more convenient than breastfeeding	20 (10)	27 (13.5)	12 (6)	53 (26.5)	88 (44)
Breastfeeding decreases mother-infant bonding	6 (3.0)	3 (1.5)	12 (6.0)	15 (7.5%)	164 (82)
It is less stressful to feed baby with infant formula than to breastfeed	15 (7.5)	26 (13)	14 (7)	43 (21.5)	102 (51)

### Practice

Table 4 shows the respondents practice of exclusive breastfeeding. While about 56% of the respondents practiced EBF, only 28.5% practiced it for up to 6 months. A quarter of respondents exclusively breastfed because they were aware of its benefits while only 7% exclusively breastfed because they preferred it. Most of the respondents (58.8%) that did not practice EBF attributed it to a busy work schedule.

**Table 4:** Practice of exclusive breastfeeding (*n* = 200)

Variable	Frequency	Percentage (%)
Practiced exclusive breastfeeding	112	56.0
Practiced EBF for up to 6 months	57	28.5
Reasons for exclusively breastfeeding child^a^		
Aware of the benefits	54	27.1
Was advised to	16	8.0
Personal preference	14	7.0
A cheaper way of feeding a child	12	6.0
Reasons for not exclusively breastfeeding child^a^		
Work schedule	117	58.8
The baby rejected breastmilk	13	6.5
Poor lactation	10	5.0
Personal preference	6	3.0
Cultural belief	3	1.5

^a^Multiple responses

### Support systems

Table 5 shows the source of breastfeeding support and the preferred source for additional support. A large number of the respondents reported that their major source of breastfeeding support came from the baby’s father (44%) and from the hospital (35.5%) while the least support came from the workplace (1.5%). The majority (74%) of the respondents indicated that they needed more support from their workplace while only about 12% felt that they needed more support from breastfeeding peer groups. Table 6 shows the availability of workplace support for breastfeeding. Only a minority of respondents indicated that they had some sort of workplace support for breastfeeding. There were limited or no designated areas for breastfeeding and insufficient or no breaks provided for breastfeeding, limited flexibility with working hours and not much breastfeeding information provided at the workplace. The duration of maternity leave provided by the workplace was only 3 months for the majority (83.5%) of respondents.

**Table 5:** Sources of breastfeeding support (*n* = 200)

	Frequency	Percentage (%)
Major source of support		
Baby’s father	88	44.0
Hospital	71	35.5
Workplace	3	1.5
Breastfeeding peer group	9	4.5
Family members	29	14.5
Preferred source for more support		
Baby’s father	66	33.0
Hospital	51	25.5
Workplace	148	74.0
Breastfeeding peer group	25	12.5
Family members	32	16.2

**Table 6:** Workplace breastfeeding support reported by respondents (*n* = 200)

Variable	Frequency (“yes” responses)	Percentage (%)
Does your workplace have designated areas for breastfeeding?	17	8.5
Does your workplace allow nursing breaks for breastfeeding employees?	19	9.5
Does your workplace allow employees to flex their work hours to accommodate breastfeeding?	19	9.6
Does your workplace provide breastfeeding information to employees?	19	9.6
Duration of maternity leave allowed by workplace		
1 month	8	4.0
2 months	8	4.0
3 months	167	83.5
4 months	8	4.0
6 months	9	4.5

## Discussion

The overall results of this survey suggest that most of the mothers working in the banks in Mainland Local Government in Lagos State had good knowledge and positive attitude towards exclusive breastfeeding. However, less than a third practised it for up to 6 months after birth. The commonest reason given for not practising EBF was a busy work schedule. Most respondents reported that they had no workplace-based support for exclusive breastfeeding.

Previous studies have highlighted the importance of workplace-based support for mothers practising exclusive breastfeeding. A study done in Nairobi demonstrated difficult work schedule as a factor associated with low practice of exclusive breastfeeding [17]. A study in Singapore revealed that although work status had no effect on the initiation of breastfeeding it did have an effect on duration of breastfeeding. Working mothers were more likely to stop breastfeeding [18]. A more recent study in Northwest Ethiopia demonstrated similar findings [19]. Common conclusions reported by these studies were the need for improved workplace-based support for breastfeeding mothers and the need to revise the two to 3 month postpartum maternity leave.

Our results are in line with previous studies demonstrating a high awareness and knowledge base concerning EBF among lactating mothers [20–23]. Our study group had a positive attitude towards exclusive breastfeeding. Previous surveys have revealed similar positive attitudes towards exclusive breastfeeding [17–19]. This result is however contrary to a previous study in which only 8 % of the respondents had a positive attitude towards EBF, although the participants in this study were undergraduate students and were younger [24].

This study is unique in that it is focused on bankers, who because of their demanding work schedule are also in need of a supportive environment so that breastfeeding can be continued and reinforced in harmony with other responsibilities, both in and out of the home. This study can be used as a knowledge base for further studies concerning breastfeeding among other working mothers.

### Limitations to this study

Despite the favourable response rate, there are important limitations to this survey. Firstly, ten questionnaires were not returned. Although this is a small number it would have been useful to know the characteristics (e.g., age, socio-economic status, etc.) of the non-responders versus the responders so as to ascertain possible reasons for the non-response. Secondly, this was purely a quantitative study with no opportunity for participants to write down comments or express other concerns and not catered for by the questionnaire. Further surveys should include a qualitative assessment and possibly interviews so as to add more information to this field of study. Thirdly, the respondents had a few hours to complete the questionnaires before collection, as opposed to more time if it had been mailed to them or collected some days later. Fourthly, the sample was limited to one group of workers (bankers), therefore results may not be generalizable to all female workers in paid employment in Lagos, Nigeria. Fifthly, the study population would have had their children at different times such that recall when answering the questionnaire would have been variable.

## Conclusion

This survey demonstrates that most of the mothers working in banks within Mainland Local Government in Lagos State have a good knowledge and positive attitude towards exclusive breastfeeding. The result of this survey highlights the importance and the need for increased workplace-based support for exclusive breastfeeding among breastfeeding mothers who work in busy employment facilities. There should be advocacy for government policy for a mandatory 6 month maternity leave for all working mothers by health workers, professional bodies and other stakeholders.

## Abbreviations

EBF: Exclusive breastfeeding
WHO: World Health Organisation
